# Computing optimal drug dosing regarding efficacy and safety: the enhanced OptiDose method in NONMEM

**DOI:** 10.1007/s10928-024-09940-9

**Published:** 2024-10-08

**Authors:** Freya Bachmann, Gilbert Koch, Robert J. Bauer, Britta Steffens, Gabor Szinnai, Marc Pfister, Johannes Schropp

**Affiliations:** 1 Department of Mathematics and Statistics, University of Konstanz, PO Box 195, 78457 Konstanz, Germany; 2 Pediatric Pharmacology and Pharmacometrics, University of Basel Children’s Hospital, Spitalstrasse 33, 4056 Basel, Switzerland; 3 ICON Clinical Research LLC, Blue Bell, PA USA; 4 Pediatric Endocrinology and Diabetology, University of Basel Children’s Hospital, Spitalstrasse 33, 4056 Basel, Switzerland; 5 Department of Clinical Research, University of Basel and University Hospital Basel, Basel, Switzerland

**Keywords:** Optimal control, State constraints, Optimal dosing, Dose optimization, Efficacy, Safety

## Abstract

Recently, an optimal dosing algorithm (OptiDose) was developed to compute the optimal drug doses for any pharmacometrics model for a given dosing scenario. In the present work, we enhance the OptiDose concept to compute optimal drug dosing with respect to both efficacy and safety targets. Usually, these are not of equal importance, but one is a top priority, that needs to be satisfied, whereas the other is a secondary target and should be achieved as good as possible without failing the top priority target. Mathematically, this leads to state-constrained optimal control problems. In this paper, we elaborate how to set up such problems and transform them into classical unconstrained optimal control problems which can be solved in NONMEM. Three different optimal dosing tasks illustrate the impact of the proposed enhanced OptiDose method.

## Introduction

OptiDose is an optimal drug dosing algorithm [1, 2] recently implemented in NONMEM [3] utilizing standard commands. This allows users to advance from modeling-and-simulation towards modeling-and-optimization by solving their own optimal dosing tasks, i.e., computing optimal drug doses with any pharmacometrics (PMX) model for a given dosing scenario. Optimal doses are associated with a model response as close as possible to a desired therapeutic goal. In the previous OptiDose implementation [2], doses are optimal with respect to efficacy, but do not account for safety. As it is essential to avoid unfavorable conditions, i.e., associated with adverse effects, both efficacy and safety need to be incorporated in the optimization.

In this paper, we enhance the OptiDose concept to include constraints on model states, so-called state constraints, to compute optimal drug doses while avoiding unfavorable conditions of the patient. Hence, the therapeutic goal is defined by a cost function and a state constraint reflecting the given efficacy and safety targets. In addition, the enhanced OptiDose method replaces the need to define the therapeutic goal by a reference function describing the time course of the desired response as in the previous OptiDose concept. Moreover, we include dosing interval optimization, which can be realized in both previous and enhanced OptiDose, to compute the optimal time point for administering the next fixed dose.

The paper is organized as follows. The “Motivation” section introduces the optimal dosing task regarding efficacy and safety along the motivational example of tumor growth inhibition [4] while avoiding neutropenia [5]. In the “Theoretical” section, the general case of a state-constrained optimal control problem and methods for solving such are presented. In the “Methods” section, necessary files and commands to realize proposed method in NONMEM are explained. The “Results” section provides relevant but substantially different examples: (i) minimizing tumor weight while avoiding neutropenia, (ii) returning an elevated biomarker to the healthy range with minimal AUC of the drug, and (iii) eradicating bacteria with minimal AUC of the drug. The paper is completed with concluding remarks in the “Discussion” section.

## Motivation

As a motivational example, we consider a tumor growth inhibition model [4] combined with a model for myelosuppression [5]. The observed time interval is [0, 32] days, the tumor grows untreatedly until day 12, and then a drug is administered daily from days 12 to 27. Four doses ${D}=({D}_1,{D}_2,{D}_3,{D}_4)$ are optimized, each dose is applied four times on consecutive days. The aim of the optimal dosing task is to reduce tumor weight (efficacy) while avoiding neutropenia (safety).

In this example, top priority is the safety target to avoid neutropenia, i.e., the neutrophil level *N* remaining above a certain threshold throughout the considered time interval, e.g.,

1 $$\begin{aligned} N(t,{D}) \ge 1 \; [10^9/L], \qquad t \in [12,32]. \end{aligned}$$

**Fig. 1:**
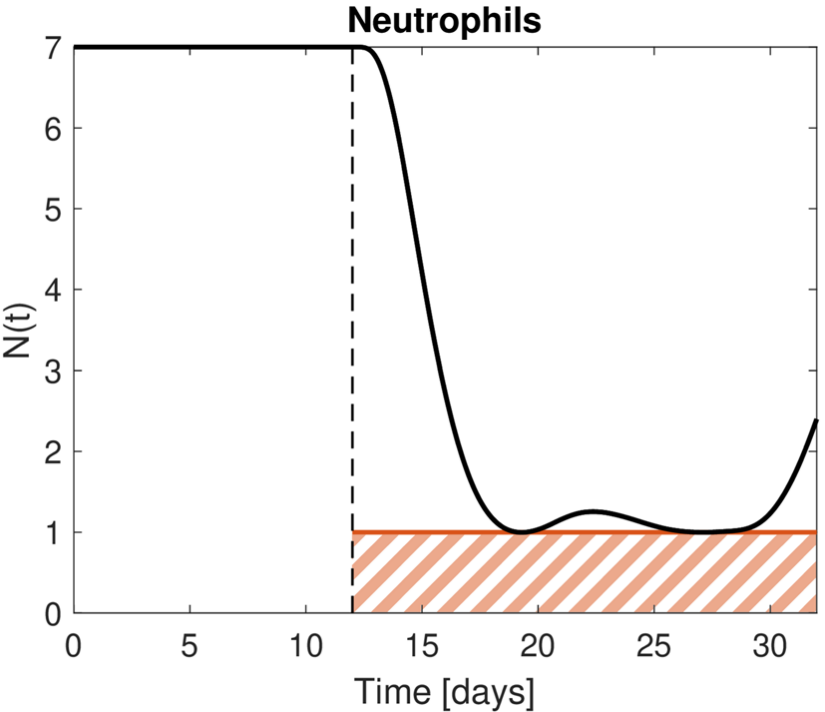
Illustration of the state constraint. The red line at $N(t) = 1$ separates feasible (neutrophils above 1, the state constraint Ineq. (1) is satisfied) from infeasible model states (neutrophils below 1, indicated as dashed red area) where the state constraint is violated. The black line shows a feasible neutrophil level over time, resulting from drug administration starting at day 12 (Color figure online)

This inequality (Ineq.) is a so-called state constraint. Model states (and associated doses) satisfying Ineq. (1) are called feasible, otherwise infeasible, see Fig. 1 for an illustration of the state constraint.

The efficacy target is to reduce tumor weight *W* as much as possible, e.g., described by minimizing the cost function

2 $$\begin{aligned} J({D}) = \int _{12}^{32} W(t,{D}) \; dt. \end{aligned}$$

The aim is to compute optimal doses regarding efficacy and safety by minimizing cost function Eq. (2) subject to state constraint Ineq. (1). Mathematically, this is a state-constrained optimal control problem (OCP).

Such a state-constrained OCP is transformed into a classical unconstrained OCP that can be solved in NONMEM as described in [2]. The idea of the transformation is to introduce a penalty function, which indirectly includes the state constraint in the optimization by measuring the violation of the state constraint, e.g.,

3 $$\begin{aligned} P({D},\rho ) = \frac{\rho }{2} \int _{12}^{32} \max \{0,1-N(t,{D})\}^2 \; dt, \end{aligned}$$

where $\rho > 0$ is a penalty parameter.^1^ The penalty function is zero if the state constraint is satisfied, and attains large values otherwise. Then, to compute the optimal doses, we minimize the sum of cost function Eq. (2) and penalty function Eq. (3), i.e.,

4 $$\begin{aligned} \min J({D}) + P({D},\rho ), \end{aligned}$$

with respect to the doses ${D}$. This can be considered as weighting the two criteria with large weight on the top priority target characterized by the state constraint.

Optimal solution for the motivational example will be discussed in the “Results” section.

## Theoretical

In this section, the general case of an optimal dosing task regarding efficacy and safety targets is presented and formulated as a state-constrained OCP. Methods for solving such are discussed.

### PMX model

Typically, a PMX model is a system of parameter- and dose-dependent ordinary differential equations,

5 $$\begin{aligned} \begin{aligned} \frac{ d}{ dt} \,y(t)&=f(t, y(t), \theta , {D}), \qquad y(t_0) = {y^0}( \theta ), \end{aligned} \end{aligned}$$

defined in the time interval $t \in [t_0,t_f]$ where $f :[t_0,t_f] \times \mathbb {R}^n \times \Theta \times \mathbb {R}^m \rightarrow \mathbb {R}^n$ describes the mechanism and *y* the model state. Further, $\theta$ denotes the model parameter in the set $\Theta$ of admissible model parameters. The doses ${D}=( {D}_1, \ldots , {D}_m ) \in \mathbb {R}^m$ are administered at fixed dosing time points according to a fixed dosing scenario. For given ${D}$, state equation Eq. (5) admits a unique solution $y({D}) :[t_0,t_f] \rightarrow \mathbb {R}^n$ (under smoothness assumptions on the right-hand side, see [1]).

Please note that Eq. (5) is a slightly simplified notation compared to [2], as we do not distinguish between PMX mechanism and inflow resulting from drug administration anymore, both are now contained in *f* in Eq. (5). Further, model parameter $\theta$ needs to be reasonably estimated, characterizing an individual or typical representative of a population, and is fixed during the optimization.

### State-constrained OCP

The aim is to compute optimal doses regarding efficacy and safety targets. Mathematically, this means minimizing a so-called cost function $J :\mathbb {R}^m \rightarrow \mathbb {R}$ subject to a state constraint,

6 $$\begin{aligned} g (y(t,{D})) \le 0, \qquad \hbox {for } t \in [t_1, t_2], \end{aligned}$$

with a function $g :\mathbb {R}^n \rightarrow \mathbb {R}$, where $t_1$ indicates the start and $t_2$ the end of the time interval where the state constraint holds. Note that $g(y(t,{D})) = 1-N(t,{D})$ recovers Ineq. (1) with $t_1 = 12, t_2 = 32$. Doses ${D}$ are called feasible, if associated model state $y({D})$ is feasible, i.e., if it satisfies the state constraint.

The state-constrained OCP reads

7 $$\begin{aligned} \min J({D}) \hbox { subject to } {\left\{ \begin{array}{ll} \, y({D}) \hbox { solves Eq.~}(5), \\ \, y({D}) \hbox { satisfies Ineq.~}(6), \\ \, \hbox {and } {D}_{min} \le {D}\le {D}_{max}, \end{array}\right. } \end{aligned}$$

where ${D}_{min}, {D}_{max} \in \mathbb {R}^m$ are a lower and upper bound on the doses, respectively.

Typically, both cost function and state constraint reflect the therapeutic goal given by efficacy and safety targets. In the motivational example, the cost function describes an efficacy and the state constraint a safety target. In other examples, as we will see in the “Results” section, this can be vice versa. The OCP structure in Eq. (7) allows for a top priority target (i.e., safety or efficacy) characterized by a state constraint, whereas the secondary target, described by an appropriate cost function, is reached as good as possible without violating the state constraint.

### Penalty methods

An approach to solve problem Eq. (7) are penalty methods [6, 7]. A state-constrained OCP is transformed into an unconstrained OCP depending on a penalty parameter. The objective function of this unconstrained OCP is given as sum of the cost function *J* of the state-constrained OCP and an additional penalty function *P*. A penalty function measures the violation of the state constraint Ineq. (6) multiplied by a penalty parameter $\rho > 0$, e.g.,

8 $$\begin{aligned} P({D},\rho ) = \frac{\rho }{2} \int _{t_1}^{t_2} \max \{0,g(y(t,{D}))\}^2 \; dt. \end{aligned}$$

The resulting unconstrained OCP then reads

9 $$\begin{aligned} \begin{aligned} \qquad&\min J({D}) + P({D},\rho ) \\&\hbox {subject to } {\left\{ \begin{array}{ll} \, y({D}) \hbox { solves Eq.~(5), } \\ \, \hbox {and } {D}_{min} \le {D}\le {D}_{max}. \end{array}\right. } \end{aligned} \end{aligned}$$

The idea of penalty methods is to solve a series of unconstrained OCPs Eq. (9) with increasing penalty parameter $\rho$, whose solutions converge towards the solution of the original state-constrained OCP Eq. (7) as $\rho \rightarrow \infty$ [7]. In the optimization in NONMEM, we fix $\rho$ to a large value.

### Evaluation of cost and penalty function

To calculate cost function value (CFV) and penalty function value (PFV), we introduce additional state variables $y_{CF}$ and $y_{PF}$, in the same way as when calculating the AUC of a drug from its concentration over time. In the motivational example, we have

$$\begin{aligned} \frac{ d}{ dt} \,y_{CF}(t)&= W(t,{D}) \; \hbox { for } t \in [t_0,t_f], \qquad y_{CF}(t_0) = 0, \end{aligned}$$

which yields the CFV

$$\begin{aligned} J({D}) = y_{CF}(t_f) = \int _{t_0}^{t_f} W(t,{D})\; dt, \end{aligned}$$

and analogously

$$\begin{aligned}&\frac{ d}{ dt} \,y_{PF}(t) = {\left\{ \begin{array}{ll} \frac{\rho }{2} \max \{0,1-N(t,{D})\}^2, &  t \in [t_1,t_2] \\ 0, &  \hbox {else} \end{array}\right. } \\  &\, \quad y_{PF}(t_0) = 0 \end{aligned}$$

providing the PFV

$$\begin{aligned} P({D},\rho ) = y_{PF}(t_f) = \frac{\rho }{2} \int _{t_1}^{t_2} \max \{0,1-N(t,{D})\}^2 \; dt. \end{aligned}$$

### Additional and other state constraints

Several state constraints can be included in the penalty method utilizing several penalty functions. Moreover, state constraints of another type may appear, e.g., some may act only at a specific time point instead of an interval, i.e., $t_1 = t_2$ in Ineq. (6). Then, the integral in Eq. (8) reduces to evaluation of the integrand at that specific time point. In the motivational example, an additional state constraint could be to demand the neutrophil level at final time $t_f$ above the threshold of 3, expressed by the penalty function

10 $$\begin{aligned} P_2(D,\rho _2) = \frac{\rho _2}{2} \max \{0, 3-N(t_f,{D})\}^2 \end{aligned}$$

and the unconstrained OCP

$$\begin{aligned} \begin{aligned} \qquad&\min J({D}) + P({D},\rho ) + P_2({D},\rho _2) \\&\hbox {subject to } {\left\{ \begin{array}{ll} \, y({D}) \hbox { solves Eq.~(5), } \\ \, \hbox {and }{D}_{min} \le {D}\le {D}_{max}. \end{array}\right. } \end{aligned} \end{aligned}$$

Another example for a different disease could be, e.g., to require a trough concentration below a certain threshold as a safety target, or a maximal efficacious concentration above a certain threshold as an efficacy target.

While the concept of penalty methods allows for several state constraints, each included with their own penalty function and penalty parameter, this increases the complexity of the problem. It might occur that the state-constrained OCP has no feasible solution, i.e., no model state satisfying all state constraints exists. In this case, the underlying efficacy and safety targets cannot be realized simultaneously.

### Augmented Lagrangian methods

In all PMX optimal dosing examples studied so far, it seemed sufficient to apply the proposed penalty method. However, a more advanced approach for solving state-constrained OCPs Eq. (7) are safeguarded augmented Lagrangian methods [7–9], which will now be briefly discussed. An additional variable $\lambda :[t_1,t_2] \rightarrow \mathbb {R}$ is introduced which approximates the Lagrange multiplier associated with the state constraint.

A popular penalty function in augmented Lagrangian methods is the Powell–Hestenes–Rockefellar [8, 10] penalty function

11 $$\begin{aligned} \begin{aligned} P_{AL}({D},\lambda ,\rho )= \frac{1}{2\rho } \int _{t_1}^{t_2}&\max \{0,\lambda (t) + \rho g(y(t,{D}))\}^2 \\  &- \lambda (t)^2 \; dt. \end{aligned} \end{aligned}$$

The unconstrained OCP reads as in Eq. (9) except for the penalty function Eq. (11) instead of Eq. (8). As with penalty methods, a series of such are solved, but multiplier approximating function $\lambda$ and penalty parameter $\rho$ are updated according to specific rules [7].

Note that choosing $\lambda = 0$ in Eq. (11) recovers Eq. (8). Thus, the penalty method, also known as Moreau–Yosida regularization in current context, can be regarded as a safeguarded augmented Lagrangian method with multiplier updates fixed to zero, see [7, Rem. 3.10]. A more elaborate multiplier update, i.e., approximating the Lagrange multiplier, might provide faster convergence [9] and improve numerical results.

## Methods

The unconstrained OCP Eq. (9) is of the same type as the OCP in [2] and can be solved in NONMEM as described in detail there and briefly summarized in the following, with few additional comments arising due to the penalty function. In addition, modifications for dosing interval optimization are discussed.

In the data file, dosing time points are indicated by AMT = 1 and final time $$t_f$$ with a dummy observation DV = 0. In the control stream, model parameters as well as penalty parameter RHO are fixed in $PK. Doses are associated with estimation parameters THETA and assigned to scale factor F=THETA since F*AMT serves as the dose in NONMEM. In $DES, PMX model equations and, if necessary, additional differential equations computing cost and/or penalty function value as discussed in the “Theoretical” section are provided. Evaluation of the maximum function is not recommended in NONMEM when computing CFV or PFV. Therefore, we utilize an IF-construction. For the motivational example, additional state variables A(12) for CFV and A(13) for PFV are as follows
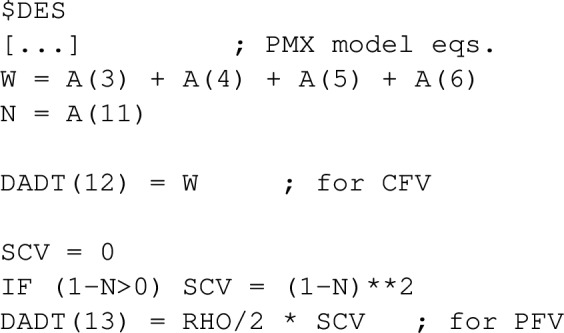
 In the $ERROR block, cost and penalty function value are computed, and the output Y is assigned to be their sum.
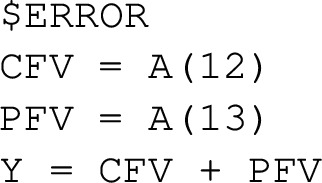


A penalty function for a state constraint acting only at a specific time point as in Eq. (10) is evaluated in the $ERROR block, e.g.,
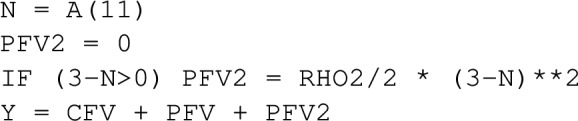


where N is the neutrophil level at final time. Other specific time points can be indicated via support points with EVID=2 in the data file, and corresponding values can be computed during $DES via IF (EVID==2)... or via IF (TIME==...). Additional support points can be utilized to report values to table, if desired. The optimization is not affected by these additional support points.

In $THETA, initial doses are specified. We recommend to start with feasible doses providing a feasible model state which does not violate the state constraint, i.e., PFV equals zero. Otherwise, a possibly large violation of the state constraint resulting in (very) large PFVs, especially for large penalty parameters, might cause numerical difficulties for the differential equation solver. Typically, there are two types of problems, either low doses or large doses satisfy the state constraint.

Several optimizations for increasing penalty parameter $\rho$ can be carried out via multiple EST -2LL runs. The accuracy can be controlled via NSIG, TOL and ATOL. If the differential equation solver struggles to integrate up to the required tolerances, try lowering ATOL first. We recommend to save values to tables and check whether PFV is (close to) zero, i.e., the state constraint is satisfied.

Several state constraints increase numerical complexity, and several penalty parameters need to be chosen. When updating penalty parameters, it is reasonable to first increase the one associated with the state constraint violated the most, until all PFVs are close to zero.

In addition, we recommend to try different (feasible) initial doses to see if there are local minima or if the global minimum is not unique, i.e., if different doses achieve the same minimal value. This can be a hint for too many doses being optimized while not enough targets to be achieved. Consequently, one should reduce the number of different doses, e.g., by administering the same dose at several time points.

Please note that the objective function value in the NONMEM output is the sum of the CFV of the original state-constrained OCP and the additional user-generated PFVs.

### Dosing interval optimization

For some applications, it might be interesting not to optimize the dose, but the dosing interval, i.e., the time point when the next dose should be administered. The proposed framework also allows one to optimize dosing intervals instead of doses. To do so, we set all dosing records to TIME=0 and specify an additional column DOSE_NR indicating the dose number in the data file, e.g., 
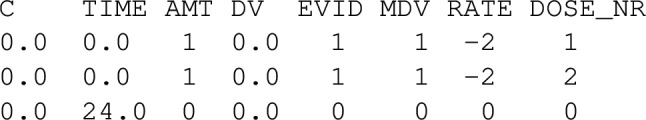
 compare the third example for an antibiotic drug in the “Results” section. RATE=-2 indicates an infusion for a fixed duration given in the control file, e.g., D1=1 for a 1 h infusion into the first compartment. The final time is 24 h indicated by a dummy observation record. In the control file, the lag parameter ALAG is utilized to delay the dosing time point, e.g., via ALAG1 = THETA*(DOSE_NR-1). Then, the first dose is administered at the fixed time point $t=0$, and the second dose is administered at time point $t=$ THETA to be optimized. The dose can be fixed either in the data file via AMT or in the control file via the scale F.

## Results

In this section, proposed enhanced OptiDose method is applied to three different optimal dosing examples regarding efficacy and safety targets in PMX arising in drug development and clinical pharmacology. Data files and control streams to set up each optimization problem in NONMEM can be found in the Appendix. Tolerances for solving the differential equation ranged from TOL = 12 to TOL = 15. For the number of significant digits, we recommend at least NSIG = 5. Both choices are problem-specific and depend on the desired accuracy of the optimal doses, the sum of CFV and PFV, and its gradient.

In all examples, for sufficiently large penalty parameter $\rho$, the optimal doses provide small gradients and PFVs close to zero, thus the state constraints are (substantially) fulfilled. All solutions were verified by the original OptiDose implementation in MATLAB [11] which was also utilized to create the figures.

### Tumor growth inhibition and myelosuppression model

First, the motivational example Eqs. (1)–(4) is considered where a tumor growth inhibition model, similar as in [4], is combined with the Friberg myelosuppression [5] model. The full NONMEM code can be found in Appendices 2 and 3. The therapeutic goal is to minimize tumor weight *W* (efficacy) given as sum of proliferating and apoptotic cells while a state constraint holds (safety, top priority), e.g., the neutrophil level *N* remains at all times above the threshold of 1.

**Fig. 2:**
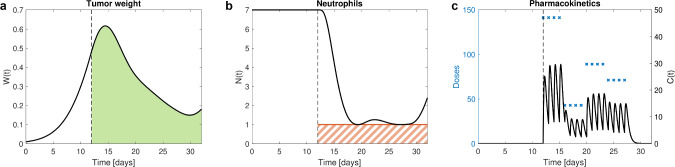
Tumor growth inhibition and myelosuppression example. In **a**, the tumor weight *W* for the optimal solution (black) is depicted, and green area represents CFV. In **b**, we see the neutrophil level *N* (black) for the optimal solution and the threshold (red) these should not fall below. The dashed red area indicates infeasible model states with undesirably low neutrophil levels. Initially, neutrophils are at their baseline of 7, shortly after drug administration starts, they drop down towards the threshold but remain above. In **c**, corresponding drug concentration *C* for optimal dosing in black and optimal doses ${D}^*$ administered via IV bolus at dosing time points (blue crosses) are displayed (Color figure online)

**Fig. 3:**
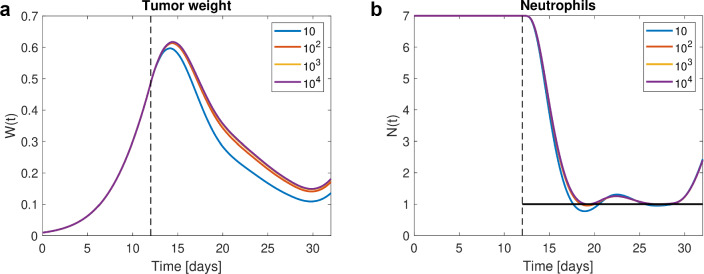
Tumor growth inhibition and myelosuppression example. For different penalty parameters, tumor weight is depicted in **a** and neutrophils in **b**. For $\rho = 10$ (blue), we observe the lowest tumor weight, however, the neutrophil level drops visibly below the threshold (black). For increasing penalty parameters, we observe numerical convergence of tumor weight and neutrophils (Color figure online)

Daily oral dosing starts at day 12 until day 27, and four doses are optimized, each applied on four subsequent days. We solve a series of unconstrained OCPs Eq. (9) with increasing penalty parameter $\rho \in \{10, 100, 1000, 10^4\}$. The initial dose is 10 for all four dose groups, providing a feasible solution with $PFV = 0$. Figure 2 shows the optimal solution for penalty parameter $\rho = 10^4$, and Fig. 3 as well as Table 1 provide a closer look at the solutions for increasing penalty parameter. We consider the solution for penalty parameter $\rho = 10^4$ with $PFV = 0.0037 \approx 0$ and $\min N = 0.9980 \approx 1$ as sufficiently good. To achieve even better compliance with the state constraint, the penalty parameter needs to be further increased. The optimal doses reduce tumor weight as much as possible without neutrophils dropping (substantially) below the threshold.

Modifying the example by including the additional state constraint $N(t_f,D) \ge 3$ as described in the “Theoretical” section via an additional penalty function Eq. (10) for penalty parameters $\rho _2 = \rho = 10^4$ lowers the fourth dose from 71.2 to 48.5 such that the final neutrophil level reaches 3.

Another interesting state constraint adopted from [12] is to limit the duration of a state variable below a certain threshold, e.g., to allow the neutrophils to drop below 1.5 for a maximum of 5 days within the whole observation interval. This duration is calculated via an additional differential equation

12 $$\begin{aligned} \frac{ d}{ dt} \,\tau (t) = {\left\{ \begin{array}{ll} 1, &  \hbox {if } N(t,{D}) < 1.5 \\ 0, &  \hbox {else} \end{array}\right. }, \quad \tau (t_0) = 0. \end{aligned}$$

The state constraint $\tau (t_f,{D}) \le 5$ is then expressed via the penalty function

13 $$\begin{aligned} P_3 ({D},\rho _3) = \frac{\rho _3}{2} \max \{0,\tau (t_f,{D}) - 5\}^2. \end{aligned}$$

**Fig. 4:**
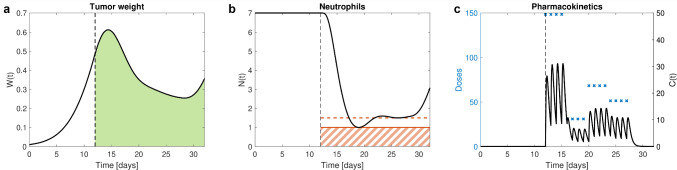
Tumor growth inhibition and myelosuppression example with additional state constraints. In **a**, the tumor weight *W* for the optimal solution (black) is depicted, and green area represents CFV. Towards the end and at final time, we observe a larger tumor weight than in Fig. 2a, due to the additional dose-limiting state constraints, and thus a larger CFV. In **b**, we see the neutrophil level *N* (black) for the optimal solution and the threshold (solid red) these should not fall below. The dashed red line is the threshold of 1.5 indicating mild neutropenia, neutrophils are below for maximal allowed 5 days. At final time, neutrophils have recovered to the level of 3. In **c**, corresponding drug concentration *C* for optimal dosing in black and optimal doses ${D}^*$ administered via IV bolus at dosing time points (blue crosses) are displayed, especially third and fourth dose are lower compared to Fig. 2c to achieve the requested higher neutrophil levels at final time (Color figure online)

A good solution (substantially) satisfying all three constraints is depicted in Fig. 4 and is found for $\rho = 10^8$ in Eq. (3) for the continuous inequality state constraint, and $\rho _2 = \rho _3 = 10^4$ in Eqs. (10) and (13) for the state constraints at final time. However, we observe the increased numerical difficulty of this problem. Different initial doses provide small differences in the optimal doses. Further, it might occur that the optimization terminates prematurely. A possible reason is the non-smoothness of the right-hand side in Eq. (12). Alternatively, instead of restricting the duration one could restrict the area below a certain threshold which enables better numerical behavior and outcomes.

**Table 1:** Tumor growth inhibition and myelosuppression example

$\rho$	$\min N$	CFV	PFV	PFV/$\rho$	$D_1$	$D_2$	$D_3$	$D_4$
10	0.774	7.95	0.406	0.0406	179.8	29.6	98.6	68.4
100	0.955	8.40	0.081	$8.1 \times 10^{-4}$	147.4	41.1	90.4	71.1
$10^3$	0.991	8.50	0.017	$1.7 \times 10^{-5}$	142.2	42.6	89.3	71.3
$10^4$	0.998	8.52	0.004	$3.7 \times 10^{-7}$	141.2	42.9	89.1	71.2

For increasing penalty parameter $\rho$, the minimal neutrophil level tends to 1 and the PFV to zero. The value PFV/$\rho$ in the fourth column measures the violation of the state constraint without penalty parameter, i.e., these values are directly comparable. For the doses, we observe a numerical convergence with increasing penalty parameter

### Biomarker indirect response model

Second, we have another look at the indirect response model from [2] where an elevated biomarker *B* shall return to the healthy target level $B_{tar}= 10$ within 14 days. Dosing is daily via IV bolus, and the dose changes weekly, providing $m=6$ doses to be optimized for an observed time interval until $t_f=42$ days.

The efficacy target is the top priority and thus formulated utilizing a state constraint, namely within 14 days the biomarker shall have reached (and then maintain) the healthy level, i.e.,

14 $$\begin{aligned} B(t,D) \le B_{tar}, \quad t \in [14,42]. \end{aligned}$$

As unnecessarily high doses lead to side effects, the secondary target (safety) is to administer as low doses as necessary, e.g., to minimize the AUC of the drug *C*. The state-constrained OCP reads

$$\begin{aligned} \min \int _{t_0}^{t_f} C(t,D) \; dt\quad \hbox {subject to} \ {\left\{ \begin{array}{ll} \hbox {PMX model,} \\ \hbox {and Ineq. }(14) \hbox { holds}. \end{array}\right. } \end{aligned}$$

**Fig. 5:**
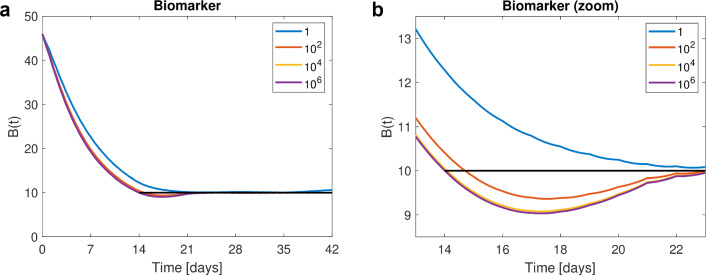
Biomarker example. For different penalty parameters, we see the biomarker levels on the full time interval in **a** and zoomed in to week three in **b.** $\rho = 1$ (blue), the state constraint (black) is still visibly violated at day 14, for increased penalty parameters, the state constraint is enforced (up to small violation). Due to the dosing scenario allowing only for weekly dose changes, for large penalty parameters, e.g., $\rho = 10^4$ (yellow) and $\rho = 10^6$ (purple), the biomarker levels reach $B_{tar}$ by day 14, but drop down even further to 9 in the third week before they approach the threshold of 10 again (Color figure online)

Optimal doses are computed for increasing penalty parameter $\rho \in \{1, 100, 10^4, 10^6\}$, each run starting from a feasible initial guess of 6 for all six doses, see Appendices 4 and 5 for full NONMEM code. Figure 5 and Table 2 display the optimal solutions for different penalty parameters.

**Table 2:** Biomarker example

$\rho$	*B*(14)	AUC = CFV	PFV	$D_1$	$D_2$	$D_3$	$D_4$	$D_5$	$D_6$
1	12.3	45.6	4.1	2.71	3.01	0.94	0.76	0.87	0.58
100	10.4	59.4	1.9	4.83	4.53	0.40	0.87	0.83	0.82
$10^4$	10.05	63.5	0.3	5.61	5.04	0.29	0.87	0.83	0.83
$10^6$	10.005	64.1	0.03	5.75	5.07	0.28	0.87	0.83	0.83
–	10.16	61.7	–	6.07	4.18	0.26	0.99	0.77	0.86

For increasing penalty parameter $\rho$, biomarker level at day 14 tends to 10 and PFV to zero. For $\rho =10^6$, ATOL was lowered to 10 instead of 12 as the differential equation solver could not integrate up to the required tolerance. The last row displays the optimal doses for the biomarker example without state constraints but a quadratic reference function describing the desired biomarker transition from elevated towards healthy, compare Example 1a in [2]. The maintenance doses as well as the sum of the two loading doses are similar

Previously [2], the therapeutic goal was characterized by a reference function describing the desired time course of the biomarker transitioning from elevated towards the healthy level. Different choices of the reference function resulted in different optimal doses [2]. The enhanced OptiDose method makes the previously mandatory reference function and discussed choices [2] obsolete.

### Antibiotic model with adaptive drug resistance

Third, a two-compartmental PKPD model with adaptive drug resistance is applied for a pediatric cancer patient under antibiotic treatment [13]. Dosing is twice daily as a 1 h IV infusion. The therapeutic goal is to minimize AUC of the drug *C* (safety) while satisfying a state constraint at final time point on the bacterial count *S* (efficacy, top priority) such as

$$\begin{aligned} S_{mean}({D}) = \frac{1}{18} \int _6^{24} S(t,{D}) \; dt\le 100, \end{aligned}$$

i.e., the mean value of *S* on the interval from 6 to 24 h shall be below the threshold of 100. The corresponding penalty function

15 $$\begin{aligned} P(D,\rho ) = \frac{\rho }{2} \max \left\{ 0,S_{mean}({D}) - 100\right\} ^2 \end{aligned}$$

is an example of the type Eq. (10). In NONMEM, we utilize an additional state variable in $DES to compute the mean value $$S_{mean}$$, and the PFV in Eq. (15) is computed in the $ERROR block. The full code can be found in Appendices 6 and 7.

**Fig. 6:**
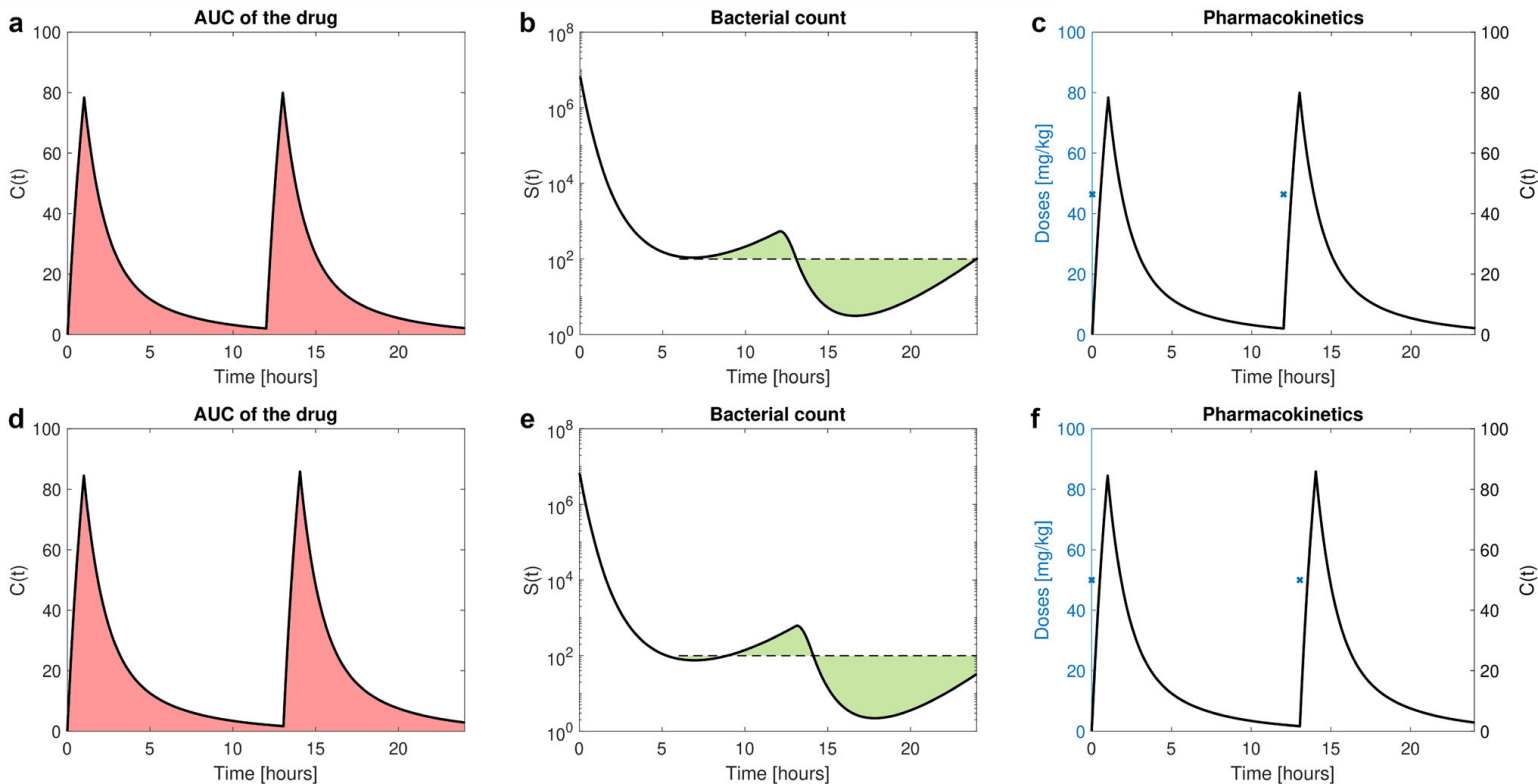
Antibiotic example. The top row shows the optimal solution for optimization of doses administered at fixed dosing time points 0 h and 12 h. The bottom row shows the optimal solution for dosing interval optimization for fixed dose of $50 \frac{mg}{kg}$. In **a** and **d**, drug concentration *C* in the central compartment for the optimal solution (black) is depicted, resulting AUC of the drug is indicated as red area. In **b** and **e**, we see the bacterial count *S* for the optimal solution and the threshold of 100 (dashed black), the mean value $S_{mean}$ is indicated as the green areas above and below the threshold (these have the same area, note the logarithmic scaling). In **c** and **f**, corresponding drug concentration in the central compartment (black) for optimal dosing and doses administered via IV infusion at dosing time points (blue crosses) are displayed (Color figure online)

The optimal dose ${D}^*=46.37 \frac{mg}{kg}$ eradicates bacteria such that $S_{mean} = 100$ with the lowest possible AUC of 419, see Fig. 6, **a** to **c**. Any lower doses would not result in sufficient bacterial eradication. The optimal daily dose $92.74 \frac{mg}{kg\cdot day}$ confirms the simulation-based recommendations of $90 \frac{mg}{kg\cdot day}$ in [13]. The optimization was carried out for different initial doses and reasonably large penalty parameters, such as $\rho \in \{10^2, 10^4, 10^6\}$, all providing the optimal dose ${D}^*$.

Modifying this example to optimize the dosing interval is displayed in Appendices 8 and 9. A fixed dose of $50 \frac{mg}{kg}$ is administered at 0 h, and the dosing interval, when the second dose is to be administered, is optimized. The optimal solution is after 13.06h which is in accordance with the previously lower dose administered after 12 h. The resulting dynamics are depicted in Fig. 6, **d** to **f**.

### Summary of results and recommendations

**Fig. 7:**
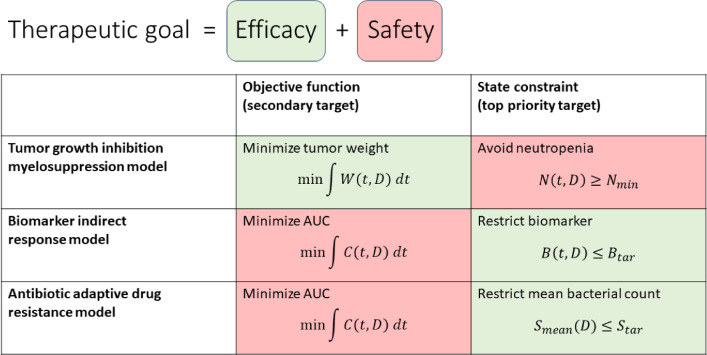
Therapeutic goals of the presented examples

In the presented approach, the therapeutic goal is given by efficacy and safety targets. In the tumor growth inhibition and myelosuppression example, the cost function describes efficacy (minimize tumor weight), and the state constraint characterizes safety (avoid neutropenia). As we have seen in the two other examples, this can be vice versa, compare Fig. 7 for a summary. The top priority is always characterized by a state constraint, whereas the secondary target, given by minimizing a cost function, is satisfied as good as possible without violating the state constraint.

It is recommended to select feasible initial doses, i.e., doses whose associated model states satisfy the state constraints. Otherwise, numerical issues may occur. Further, we recommend to try different initial doses in general, eventually to re-optimize (i.e., to utilize optimal doses as initial doses and re-run NONMEM file), and to always check whether PFVs are close to zero. In case of non-uniqueness issues, i.e., different solutions providing similar OFVs, we recommend to reduce the degree of freedom, e.g., by reducing the number of administered doses by grouping doses, i.e., by repeatedly administering the same dose, or by including additional desired targets. Similar considerations apply to optimizing dosing intervals.

## Discussion

Optimal control is an emerging discipline in PMX [14], with pioneering work in the field of optimal dosing, e.g., by Jönsson et al. [15] or Iliadis et al. [16]. Especially in infectious diseases [17–19] and oncology [12, 20–23], optimal control has been applied in the past.

The present paper is the most recent one in a series of three utilizing our OptiDose concept to compute optimal drug dosing regimens. In the first paper [1], the OptiDose concept to compute optimal drug doses for PMX models was introduced and realized in MATLAB [11]. The major novelty of the OptiDose concept was to directly optimize the drug doses which generate the drug concentration and to quantify the therapeutic goal utilizing a reference function. The aim of the second paper was to realize the OptiDose concept in NONMEM, see [2]. Based on a reformulation of the OptiDose optimal control problem as an optimization problem, the optimal dosing task can be solved efficiently in NONMEM utilizing standard commands.

In the present paper, the OptiDose concept in NONMEM was enhanced to include efficacy and safety targets. While an easy approach could be to add all targets into one cost function, this might not provide satisfying solutions in situations with a top priority target that needs to be satisfied, e.g., as to avoid neutropenia in cancer treatment, and a secondary target which is achieved as good as possible. Mathematically, this leads to a state-constrained optimal control problem, i.e., to optimizing a cost function characterizing the secondary target while an inequality state constraint, separating feasible from infeasible model states, to describe the top priority target, holds. In the present paper, we solve such by applying a penalty method, which can be seen as a simplified augmented Lagrangian method [7]. The developed enhanced OptiDose method enables users to compute optimal drug dosing with respect to efficacy and safety targets in NONMEM utilizing standard commands.

The impact of this approach was demonstrated by solving three substantially different optimal dosing examples with respect to efficacy and safety. In particular, the solutions computed for the tumor growth inhibition and myelosuppression example (see Figs. 2 and 4) display, how a precise treatment of an individual patient for prescribed efficacy and safety targets could look like. We achieve the maximal tumor reduction without entering the life-threatening condition of neutropenia, thus answering the question: How far can we go, how much can the patient still tolerate? Finding such solutions without optimal control, i.e., based on numerous simulations for varying dosing strategies, is difficult and laborious.

A prerequisite for solving optimal dosing tasks is a predictive model including reasonably estimated model parameters. Model parameters are fixed during the optimization and can either characterize an arbitrary (eventually extreme) individual or typical representative of a population. In the presented examples, model parameters were assumed to be sufficiently well estimated from data. Further, a sensitivity analysis on the optimal doses allows one to investigate how uncertainty in the model parameters propagates to the optimal doses. In clinical practice, such as in therapeutic drug monitoring, individual model parameters can be (re-)estimated from individual measurements and applied to the proposed method to optimize doses for a particular individual.

## Data Availability

No datasets were generated or analysed during the current study.

## Appendix 1

**Model equations of the three PMX examples**

*Tumor growth inhibition myelosuppression model*

We combine the Friberg [5] myelosuppression model and an adapted Simeoni [4, 24] tumor growth inhibition model. The tumor weight *W* is given as the sum of proliferating tumor cells $T_{prol}$ and different stages of apoptotic tumor cells $T_{ap1}, T_{ap2}, T_{ap3}$. Maturing of neutrophils is modeled with proliferating cells $N_{prol}$, three transit compartments $N_{tr1}, N_{tr2}, N_{tr3}$ and circulating neutrophils *N*.

$$\begin{aligned} \frac{ d}{ dt} \,Abs&= In(t,D) - k_a Abs, \\ \frac{ d}{ dt} \,A_C&= k_a Abs - k_{el} A_C, \\ C&= \frac{A_C}{V}, \\ W&= T_{prol} + T_{ap1} + T_{ap2} + T_{ap3}, \\ \frac{ d}{ dt} \,T_{prol}&= \frac{2 \lambda _0 \lambda _1 T_{prol}^2}{(\lambda _1 + 2 \lambda _0 T_{prol}) W} - \frac{E_{max,T} \, C}{EC_{50,T}+C} T_{prol}, \\ \frac{ d}{ dt} \,T_{ap1}&= \frac{E_{max,T} \, C}{EC_{50,T} + C} T_{prol} - k_1 T_{ap1}, \\ \frac{ d}{ dt} \,T_{ap2}&= k_1 (T_{ap1} - T_{ap2}), \\ \frac{ d}{ dt} \,T_{ap3}&= k_1 (T_{ap2} - T_{ap3}), \\ \frac{ d}{ dt} \,N_{prol}&= k_{prol} N_{prol} \left( 1 - \frac{E_{max,N} \, C}{EC_{50,N} + C} \right) \left( \frac{Circ^0}{N} \right) ^{\gamma } \\&\quad - k_{tr} N_{prol}, \\ \frac{ d}{ dt} \,N_{tr1}&= k_{tr}(N_{prol} - N_{tr1}), \\ \frac{ d}{ dt} \,N_{tr2}&= k_{tr}(N_{tr1} - N_{tr2}), \\ \frac{ d}{ dt} \,N_{tr3}&= k_{tr}(N_{tr2} - N_{tr3}), \\ \frac{ d}{ dt} \,N&= k_{tr} N_{tr3} - k_{circ} N. \end{aligned}$$

Initial values are given as $T_{prol}(0) = W^0$, $N_{prol}(0) = N_{tr1}(0) = N_{tr2}(0) = N_{tr3}(0) = N(0) = Circ^0$, all others are zero. Parameter values chosen were $V=2.79$, $k_a = 5$, $k_{el} = 2.53$, $\lambda _0 = 0.194$, $\lambda _1 = 0.246$, $k_1 = 0.666$ from [4] with an Emax-effect term with $E_{max,T} = 2$, $EC_{50,T}=80$ instead of previous linear effect term. Initial tumor weight is $W^0 = 0.0098$ which grows unperturbed until drug administration starts on day 12. Further, parameter values for the myelosuppression model were chosen as $Circ^0 = 7$, $E_{max,N} = 1$, $EC_{50,N} = 50$, $k_{prol} = k_{tr} = k_{circ} = 2$, $\gamma = 0.1$. The combination of these models and choice of parameter values is for illustrational purposes and does not reflect a real patient.

*Biomarker indirect response model*

$$\begin{aligned} \frac{d}{dt} A= &   In(t,{D}) - k_{el} A, \qquad \qquad \qquad \qquad \; \, \, A(0) = 0,\\ \frac{d}{dt} B= &   k_{in} - k_{out} \left( 1 + \frac{E_{max} C}{EC_{50} + C} \right) B, \qquad B(0) = B^0, \end{aligned}$$

with $$C = \frac{A }{V}$$ and parameter values $$ V = 3$$, $$B^0= 46$$, $$k_{out} = 0.02$$, $$k_{in}=0.92$$, $$k_{el}=0.49$$, $$E_{max}=8.8$$, $$ {EC}_{50}=0.81$$ [2].

*Antibiotic adaptive drug resistance model*

$$\begin{aligned} \frac{ d}{ dt} \,A_1&= In(t,D)- (k_{el} + k_{12}) A_1 + k_{21} \, A_2, \\ \frac{ d}{ dt} \,A_2&= k_{12} \, A_1 - k_{21} \, A_2,\\ \frac{ d}{ dt} \,S&= K_g \left( 1- \frac{S}{S_{max}}\right) S - \frac{E_{max} \, C_p}{AD \cdot EC_{50} + C_p} S, \end{aligned}$$

for $$k_{el} = \frac{Cl}{V_1}$$, $$k_{12} = \frac{Q}{V_1}$$ and $$k_{21}=\frac{Q}{V_2}$$ with unbound concentration $$C_p =0.9 \, \frac{A_1}{V_1}$$ and adaptive resistance factor $$AD = 1 + \beta (1-\exp (-\alpha C_p \cdot t))$$. Initial amounts are zero and initial bacterial count is $$S(0) = S^0$$. Parameter values for a typical 20 kg individual are $$Cl = 4.33$$, $$V_1 = 8.63$$, $$Q = 1.66$$, $$V_2 = 4.26$$ obtained from allometric scaling, and $$K_g = 1.04$$, $$S^0 = 10^{6.8}$$, $$S_{max}=10^{9.41}$$, $$E_{max} = 9.38$$, $$ {EC}_{50}= 3.49$$, $$\alpha = 0.0143$$, $$\beta = 29.2$$ [13].

## Appendix 2

**Tumor growth inhibition + myelosuppression example. The data file** tgimyelo.dat **.**

## Appendix 3

**Tumor growth inhibition + myelosuppression example. The control file** tgimyelo.ctl **.**

## Appendix 4

**Biomarker example. The data file** biomarker.dat **.**

## Appendix 5

**Biomarker example. The control file** biomarker_sc.ctl **.**

## Appendix 6

**Antibiotic example. The data file** antibiotic.dat **.**

## Appendix 7

**Antibiotic example. The control file** antibiotic.ctl **.**

## Appendix 8

**Antibiotic interval example. The data file**antibiotic_interval.dat.

## Appendix 9

**Antibiotic interval example. The control file** antibiotic_interval.ctl.

## Footnotes

1 Rho (lower case $\rho$) is the 17th letter of the Greek alphabet.
